# Evolving use and indications for implantable cardioverter defibrillators

**DOI:** 10.1007/s12471-018-1193-2

**Published:** 2018-11-13

**Authors:** J. R. de Groot

**Affiliations:** Department of Cardiology, Amsterdam University Medical Centers/University of Amsterdam, Heart Center, Amsterdam, The Netherlands

Since the appreciation of ventricular arrhythmias as a major cause of sudden cardiac death (SCD), enormous efforts have been undertaken to provide a curative treatment for this condition. In the 1970s and 1980s, much was invested to suppress ventricular arrhythmias with antiarrhythmics. This field of therapy came to an abrupt end when the CAST and SWORD studies were prematurely terminated because of proarrhythmia and increased mortality in patients treated with Class IC antiarrhythmics and d‑sotalol respectively [1, 2]. Subsequently, large randomised clinical trials demonstrated that mortality can be reduced by treating SCD with an implantable cardioverter defibrillator (ICD) in patients with a diminished left ventricular ejection fraction, both in primary and secondary prevention settings [3, 4]. Hence, although not preventing the cause or the event of arrhythmia, these devices successfully treat its lethal consequences. The practice of ICD implantation has grown since, to approximately 6,000 implantations yearly in the Netherlands alone. Meanwhile, indications for ICD implantation have been refined, and modern devices are equipped with algorithms that may detect clinical deterioration and atrial arrhythmias early or prevent inappropriate or unnecessary shock therapy. During the same time period, automatic defibrillators have increasingly entered the public space to reduce mortality of out-of-hospital cardiac arrest.

This issue of the Netherlands Heart Journal focuses on contemporary cardiac implantable electronic device (CIED) therapy, and features a number of manuscripts that delineate how both implanted and external device therapy is being provided to optimise the balance between benefit and harm. In a series of paediatric and adolescent ICD recipients (defined as <25 years of age and consisting of mainly adolescents, with median age of 16.5 and 19.0 years and median length of 1.70 and 1.83 m respectively), Quast et al. compare the rate of complications between transvenous ICDs (TV-ICDs) and subcutaneous ICDs (S-ICDs) [5]. This was a retrospective analysis in which, as the authors admit, an era effect was detectable: follow-up duration was 60 months in 46 TV-ICD patients versus 40 months in 35 S‑ICD patients. In other words, in recent years S‑ICDs were implanted more in this patient category. Quast et al. demonstrate that there were more lead-related complications (23% versus 0% in the TV-ICD and S‑ICD group respectively, *p* = 0.02), whereas there was no difference in infection (2% versus 10%) or the occurrence of appropriate (25% versus 27%) or inappropriate (22% versus 14%) therapy. Hence, the total number of device-related complications was similar between the groups, although the nature of these complications differed.

Atrial fibrillation (AF) increases the risk of stroke, mortality and heart failure, and may complicate ICD therapy through the triggering of inappropriate shocks. Baalman et al. describe the design of the forthcoming multicentre INDICO-AF study, that aims at understanding the epidemiology and pathophysiology of new-onset AF in patients with coronary artery disease, diminished left ventricular ejection fraction and a primary prevention indication for ICD implantation [6]. This study will take advantage of the incorporation of a novel algorithm in single-chamber ICDs that detects AF in a similar manner as implantable loop recorders do, that is, by analysis of the Lorentz plot of R‑R intervals. As currently both the incidence and prevalence of AF in this patient category is not well described, and markers for the development of AF are lacking, INDICO-AF may shed light on which patients may need further vigilance for AF detection, and refine patient selection for the use of such algorithms. This is irrespective of whether patients with device-detected, but not ECG-documented, AF need immediate anticoagulation based on their CHA_2_DS_2_VASc (congestive heart failure, hypertension, age ≥75 years [doubled], diabetes mellitus, prior stroke [doubled]-vascular disease, age 65–74, sex category) score, which is subject of debate and currently being investigated in the randomised NOAH-AF and Artesia studies [7, 8]; detection of AF in patients with an ICD may at least allow measures to be taken to prevent inappropriate shocks.

Inappropriate shocks in S‑ICDs frequently relate to oversensing of the T wave, and can be prevented by vector testing during exercise or updates of the detection software [9]. The latter is described by Larbig et al. who investigated 139 S‑ICD patients in a single-centre study [10]. They demonstrate that the incidence of T‑wave oversensing was significantly lower in the newer generation S‑ICDs (4.2% versus 15.4%, *p* < 0.05), and that updates of the detection software in the older models was associated with a similar reduction in T‑wave oversensing from 13.4% to 4.6% (*p* = 0.07). Also here, as in the study of Quast and colleagues, there may have been an era effect, and increasing experience with the device over time may have improved patient selection, and reduced inappropriate shocks.

Not only has there been a tremendous increase in the practice of implantable defibrillator therapy, but also in the availability of automated external defibrillators (AEDs) in the public area. Nas et al. report on a prospective registry on out-of-hospital cardiac arrest, and compare the primary outcomes of survival with discharge and return of spontaneous circulation in this registry (data collected between 2013 and 2016) with data from historical controls (2008–2011) in the same area. The study population was restricted to patients transported to the Radboud UMC in Nijmegen, and arrests witnessed by emergency medical services were excluded from the analysis. In the study cohort, bystander cardiopulmonary resuscitation was performed more frequently than in the historical controls (78% versus 63% respectively, *p* < 0.001) and AED was used in a higher proportion of patients (46% versus 23% respectively, *p* < 0.001). Over time, there was a shift from shocks delivered by emergency personnel (76% in 2008–2011 versus 59% in 2013–2016, *p* < 0.001) toward shocks delivered by AED (15% in 2008–2011 versus 39% in 2013–2016, *p* < 0.001). Despite no difference in proportion of patients with return of spontaneous circulation, survival was higher in the study cohort than in the controls. In both the historical cohort and the study cohort, bystander CPR and AED use were independently associated with survival after out-of-hospital cardiac arrest.

Pacemaker implantation as a consequence of atrioventricular block following transcatheter aortic valve implantation is a relatively common complication, particularly encountered in the earlier valve generations. Gonska and colleagues investigate whether pacemaker implantation in this setting is associated with an altered one-year prognosis [11]. Using balloon-expandable ES3, mechanically expanded Lotus and self-expandable CoreValve devices, they report a pacemaker implantation rate of 24.4%, which seems rather high for a contemporary cohort. Although there were more major bleeding events in patients with a pacemaker, there was no difference in mortality (12.2% versus 12.5%, *p* = 0.94) or major adverse events (24.5% versus 22.1%, *p* = 0.55) at one-year follow-up between patients with and patients without pacemaker implantation. This was also the case for patients with a left ventricular ejection fraction lower than 45%.

Until recently, implantation of a pacemaker or ICD implied life-long abstinence from magnetic resonance imaging (MRI). However, newer devices are fully or partly compatible with whole-body MRI, although precautions should be taken. In their point-of-view article, Maass, Hemels and Allaart detail how MRIs can be safely performed in device-carrying patients, and discuss precautions to be taken with respect to pre-imaging programming and trouble-shooting of unanticipated complications or adverse events [12].

Together, the contributions to this issue of the Netherlands Heart Journal present a contemporary overview of where we are moving with respect to indication, use, and handling of ICDs and pacemakers.
